# Let us unite against COVID-19 – a New Zealand perspective

**DOI:** 10.1017/ipm.2020.44

**Published:** 2020-05-14

**Authors:** G. Bandyopadhyay, A. Meltzer

**Affiliations:** 1 Whanganui DHB, Whanganui, New Zealand; 2 University of Otago, New Zealand; 3 Gonville Medical Centre, WRHN, Whanganui, New Zealand

**Keywords:** Coronavirus, COVID-19, mental health, New Zealand

## Abstract

Novel coronavirus 2019 (COVID-19) has shaken the existence of mankind worldwide, including that of New Zealand. In comparison to other countries, New Zealand has had a very low number of confirmed and probable cases as well as COVID-19-related deaths. New Zealand closed its borders and rapidly declared a stringent lockdown to eliminate COVID-19. The country’s ‘go hard, go early’ policy serves as an exemplar for the rest of the world to date. The mysterious nature of COVID-19 has caused tremendous stress and uncertainty leading to universal conflict between public health and state economy. Mental health services and non-government organisations have been proactive in the fight against COVID-19. Though there has been no significant rise in referrals to secondary mental health services to date (4 May 2020), a rapid surge in mental health presentations is widely anticipated. Telehealth may prove to be an efficient and cost-effective tool for the provision of future health services.

## Introduction

The global pandemic of novel coronavirus 2019 (COVID-19) has claimed hundreds of thousands of lives and disrupted social, psychological and economic rhythms worldwide. In response to the pandemic, New Zealand has closed its borders and declared a stringent lockdown to eliminate the virus. This has resulted in low numbers of confirmed and probable cases as well as COVID-19-related deaths. New Zealand’s ‘go hard, go early’ policy has served as an exemplar for the rest of the world to date (Robertson, 2020). Regardless, the uncertain nature of this highly contagious virus has had significant impact on human emotion, behaviour, cognition and well-being requiring special consideration.

## New Zealand’s health structure and demographic profile

Health and Disability Services in New Zealand are delivered by a complex network of organisations. Approximately one-fifth of the government’s spending goes to the health sector. Health funding is administered by 20 District Health Boards that are both purchasers and providers of health services including primary care, hospital, public health and elderly services as well as non-government organisations (NGOs) including Maori and Pacific providers. General Practice visits and pharmaceuticals require co-payments but are heavily subsidised. Government-funded health services are free to New Zealanders (Ministry of Health New Zealand, 2017).

The majority of New Zealand’s population of 4.9 million is of European descent (72%), followed by Maori (16.5%), Pacifica (9.5%), Asian (5%), African (1.5%) and Other (1%). Over a quarter of the New Zealand population was born overseas and one-fifth are over 65 years of age (Statistics of New Zealand, 2019). Maori and elderly populations suffer the greatest health and social disparities (Ellison-Loschmann *et al*. 2006). Thus, these groups face the highest risk for disastrous effects of coronavirus.

## Mental health care in New Zealand

One in five New Zealanders experiences mental health issues every year, and the annual cost of serious mental health is estimated at 5% of gross domestic expenditure. New Zealand has persistently high suicide rates, with youth suicide rates among the worst of the OECD (Organisation for Economic Co-Operation and Development) countries (He Ara Oranga, 2018).

In 2019, the New Zealand government introduced the Mental Health and Wellbeing Commission Bill to the Parliament based on the Report from the Government Inquiry into Mental Health and Addiction (He Ara Oranga, 2018). A record 1.9 billion NZD was allocated to mental health over a period of 5 years with the aim of integrating mental health services and primary care (Mental Health and Wellbeing Commission, 2019). Despite this, it may soon be challenging to provide adequate service to our most vulnerable as a rapid surge of mental health issues is expected in response to COVID-19.

## Timeline of COVID-19 in New Zealand

The coronavirus outbreak started in December 2019 in Wuhan City, China, where cases of mysterious illness including pneumonia of unknown origin were noted. On 3 January 2020, New Zealand’s government placed entry restrictions on those transiting through or travelling from mainland China requiring a 14-day period of self-isolation upon return to the country. New Zealand’s Ministry of Health set up a team to monitor the situation on 24 January 2020, and risk of viral epidemic was considered low at that time (Ministry of Health, 2020 *b*).

On 28 February 2020, New Zealand reported its first case of COVID-19 in an adult male travelling from Iran. From 14 March, the 14-day self-isolation mandate was extended to anyone entering the country with the exception of those travelling from the Pacific Islands. In an unprecedented turn of events, all borders and entry points were closed to non-residents on 19 March 2020. On 21 March, a four-level COVID-19 alert system was introduced to specify public health and social measures to progressively move the country through phases to prepare, reduce, restrict and lockdown (New Zealand Government, 2020 *b*). The alert was initially set at level 2 (reduce), but was subsequently raised to level 3 (restrict) on the afternoon of 23 March. A three-month Epidemic Notice was declared on the same day as a public policy tool to help government agencies to act swiftly and effectively in a rapidly evolving crisis situation (Ministry of Health New Zealand, 2020 *b*). Beginning at 11:59 pm on 25 March, the alert level was moved to level 4, putting the country into one of the world’s most stringent nationwide lockdowns until 11:59 on 27 April 2020. At that time, the nation returned to level 3 for an estimated further 2 weeks (New Zealand Govt, 2020 *b*). Education, business and travel have been severely impacted under alert levels 3 and 4.

To date (4 May 2020), New Zealand has had a total of 1,487 confirmed and probable cases with zero new cases recorded in the past 24 hours. In total, 1276 people have recovered (86%) and only 4 people are in hospital. At the time of writing, the age groups most frequently infected by COVID-19 are 20–29 (24%), followed by 50–59 (16%), 40–49 (15%) and 30–39 (15%). Sadly, 20 people have lost their lives (Statistics New Zealand, 2020). All were over 60 years of age with underlying medical conditions (Ministry of Health New Zealand, 2020 *a*). Seventy percent of all New Zealand COVID-19 mortalities have been linked to aged care facilities (Beynen, 2020). Despite their vulnerability, those aged 70 years or over account for only 7% of total cases demonstrating the protective outcomes of New Zealand’s approach to COVID-19 containment (Ministry of Health New Zealand, 2020 *a*).

## Psychological impact of COVID-19

The mysterious nature of COVID-19 combined with a lack of evidence-based treatment to date has caused tremendous stress, turmoil and uncertainties worldwide. COVID-19 has threatened not only physical health but emotional and cognitive well-being as well. Faced with a perceived threat of infection, people begin to regard each other with suspicion and display avoidant behaviours (Slovic, 1987). The deleterious effect of quarantine including frustration and boredom puts vulnerable people with pre-existing medical conditions, mental illness and advanced age at risk of further psychological morbidity (Brooks *et al*. 2020). Fear of death, anxiety, panic attacks, adjustment disorders, depression, obsessive compulsive disorder, post-traumatic stress disorder and relapse of pre-existing mental illness are predicted to escalate (Chatterjee *et al.* 2020). Those with disabilities, developmental disorders and dysfunctional backgrounds face additional risks (Brooks *et al.* 2020). There has been a 20% increase in reported domestic violence as victims face increasing difficultly escaping their perpetrators whilst in lockdown (Johnston, 2020).

Parents of young children are increasingly stressed as New Zealand has announced plans for prolonged online schooling (Cluver *et al*. 2020). Children with Attention Deficit Hyperactivity Disorder, Autistic Spectrum Disorder and other developmental disorders and disabilities appear to be struggling while those with social anxiety seem to thrive in the context of reduced face to face social interaction. Regardless, a surge of mental health presentations in children is expected as New Zealand exits level 4 lockdown.

There is a high level of anxiety amongst health care professionals in regard to limited resources. Many doctors hailing from overseas have returned to their countries of origin, causing significant shortages of frontline health care professionals (Whanganui DHB, 2020 *a*). Those remaining face concerns around access to personal protective equipment, ventilators and clear cut isolation policies (Shanafelt *et al.* 2020). Health care workers appear to be at risk of clinical burnout and stigmatisation (Brooks *et al.* 2020).

New Zealand has only just emerged from 33 days of alert level 4 lockdown where all non-essential services were closed. Many businesses remain unable to operate under alert level 3. Approximately 20% of the population faces imminent unemployment (Taunton, 2020). There are significant concerns about deleterious social and economic consequences.

Clear and consistent public communication has been essential during periods of psychological turmoil. Several initiatives by government and non-government agencies have been launched to support people during the COVID-19 pandemic. Well-communicated public education programmes have helped to create a sense of public trust (Wallis, 2020).

## Authors’ observations

Despite recent increases in phone calls from distressed patients and families, to date, there has been no significant increase in secondary mental health referrals. As with other medical services, psychiatric inpatient admissions decreased across all age groups during the level 4 lockdown (Whanganui, 2020 *b*). There is concern that patients are delaying medical care due to fears of COVID-19 (Brooks *et al.* 2020). Post disaster surges in mental health presentations are anticipated in line with historical data (Neria *et al.* 2008). Mental health professionals aim to continue ‘business as usual’ primarily by virtual consultation supplemented by face to face assessment where indicated (Whanganui, 2020 *b*).

## Some positives

As COVID-19 has emerged in New Zealand, health care providers have had to adapt their patient consultation style. Telehealth and e-prescribing have rapidly become the new normal, reducing barriers to care (NZ Telehealth Forum and Resource Centre, 2020). Telehealth, teleconferencing and webinars have emerged as efficient and cost-effective tools in health care services (Huesch, 2013). However, further research is needed regarding long-term telehealth outcomes.

New Zealanders have championed the art of ‘staying together, whilst staying apart’. Social media and online platforms have brought together friends and families living miles apart. There has been a surge of good Samaritans quietly supporting the frail and elderly during these difficult times. Teddy bears peak out from household windows to lift the spirits of our children. Despite the restraints of social distancing, New Zealanders have remained connected, raised funds and entertained others just to say ‘we care’.

New Zealand has survived many man-made and natural disasters over the years including several volcanic eruptions, the 2011 Canterbury earthquakes and the 2019 hate fuelled shootings in Christchurch. Just as we did then, we are sure the nation will stay strong and will in fact be strengthened by our collective experience.

Working together and supporting each other, we will get through this.
